# The Rubber Hand Illusion: Top-down attention modulates embodiment

**DOI:** 10.1177/17470218221078858

**Published:** 2022-02-24

**Authors:** Rémi Thériault, Mathieu Landry, Amir Raz

**Affiliations:** 1 Department of Psychiatry, McGill University, Montréal, Québec, Canada; 2 Department of Psychology, Université du Québec à Montréal, Montréal, Québec, Canada; 3 Institute for Interdisciplinary Brain and Behavioral Sciences, Chapman University, Orange, CA, USA; 4 Integrated Program in Neuroscience, Montreal Neurological Institute, Montreal, Quebec, Canada; 5 The Lady Davis Institute at the SMBD Jewish General Hospital, Montréal, Québec, Canada

**Keywords:** Rubber hand illusion, top-down cognition, bottom-up processes, body ownership, consciousness, selfhood

## Abstract

The Rubber Hand Illusion (RHI) creates distortions of body ownership through multimodal integration of somatosensory and visual inputs. This illusion largely rests on bottom-up (automatic multisensory and perceptual integration) mechanisms. However, the relative contribution from top-down factors, such as controlled processes involving attentional regulation, remains unclear. Following previous work that highlights the putative influence of higher-order cognition in the RHI, we aimed to further examine how modulations of working memory load and task instructions—two conditions engaging top-down cognitive processes—influence the experience of the RHI, as indexed by a number of psychometric dimensions. Relying on exploratory factor analysis for assessing this phenomenology within the RHI, our results confirm the influence of higher-order, top-down mental processes. Whereas task instruction strongly modulated embodiment of the rubber hand, cognitive load altered the affective dimension of the RHI. Our findings corroborate that top-down processes shape the phenomenology of the RHI and herald new ways to improve experimental control over the RHI.

Feelings of owning your body and limbs—body ownership—represent a core component of our conscious experiences. This central feature follows from the significant amount of interoceptive and exteroceptive sensations processed by the brain, while our bodies provide the main perspective from which we engage our environment and the emergence of selfhood and personal identity (de Vignemont, 2011). Importantly, the phenomenological experience of owning your body follows from complex neural processes that include multisensory integration (de Vignemont, 2011; Longo et al., 2008; Tsakiris, 2010). Grounded in this overarching framework, the current research investigates whether higher-order cognition exerts a top-down influence over the Rubber Hand Illusion (RHI)—a phenomenological distortion of body ownership (Botvinick & Cohen, 1998). In the standard approach to the RHI, participants’ real hands are occluded from their view and replaced with a clearly visible fake rubber arm positioned so as to mimic their actual limb. Stroking the visible fake hand and the occluded real hand simultaneously in this context yields peculiar feelings of ownership over the fake arm. The synchronicity of visual and tactile sensations therefore leads to inferences that skew feelings of body ownership to the point of incorporating the fake rubber hand (Riemer et al., 2019).^1^

In recent years, a growing body of research has focused on the mechanisms underlying the emergence of these body distortions to inform the phenomenology of selfhood and embodiment (Kilteni et al., 2015; Tsakiris, 2010, 2017). This work shows that body ownership represents an intrinsic component of our conscious experiences, as the coupling between sensorimotor processes and the environment shapes how we interface with the world (de Vignemont, 2011; Hurley, 1998). Thus, our body seemingly represents an extension of the conscious mind, while the RHI emphasises the malleability of these representations. Prevailing views often argue that the unusual experience of the RHI proceeds from inferential processes that integrate somatosensory, proprioceptive, and visual inputs (Armel & Ramachandran, 2003; Botvinick & Cohen, 1998; Samad et al., 2015; Seth, 2013). The attribution of tactile sensations to the fake arm seemingly proceeds from temporal alignment with visual inputs, which entails that the influence of sight on the RHI is contingent on temporal coupling between sensations (Shimada et al., 2009, 2014).

Beyond these core components, ongoing research further highlights the influence of top-down factors, thus departing from a wholly bottom-up account (Dempsey-Jones & Kritikos, 2014; Kilteni et al., 2015). For example, inconsistencies between the position of the rubber hand and internal representations of the real limb’s actual posture impair the RHI, thus showing that prior information shapes the emergence of this phenomenon (Ehrsson et al., 2004). Likewise, handedness, anatomy, texture, incorporeability, affect, and awareness of internal body signals can also modulate the illusion to some extent (Dempsey-Jones & Kritikos, 2017, 2019; Kilteni et al., 2015; Tsakiris, 2010, 2017). The idea that top-down factors can influence the RHI is reminiscent of findings that similarly feature the involvement of top-down suggestions in the context of other multimodal sensory integration phenomena, such as the McGurk effect (e.g., Déry et al., 2014; Lifshitz et al., 2013; McGurk & MacDonald, 1976), and therefore echoes recent discussions about multisensory integration more generally (Hartcher-O’Brien et al., 2017).

The current work aims to expand this research trajectory by investigating the impact of top-down factors over the RHI. Our experimental approach follows from previous work on the decomposition of embodiment into different latent constructs via an established questionnaire and factor analysis, including feelings of embodiment (Longo et al., 2008). Embodiment refers to the idea that the body shapes our psychological processes, whereby the features of the human mind are predicated upon the features of the human body. We therefore intended to use a similar model for the assessment of top-down factors onto the different dimensions of body ownership in the RHI. Our strategy was twofold. First, we altered the availability of cognitive resources using a working memory load manipulation—a standard experimental approach to examine the involvement of higher-order cognitive resources in psychological phenomena (e.g., Bodner & Stalinski, 2008; Fahey et al., 2018). This experimental manipulation assesses the capacities of higher-order cognition, such as voluntary attention or working memory, by diverting cognitive resources away from the primary task and towards a secondary somewhat difficult one (Gazzaley & Nobre, 2012). Resource limitations therefore arise when performance suffers from having to engage both tasks simultaneously.

Working memory contributes to filtering certain sensory stimuli at an early processing stage of processing, as revealed by corresponding changes in precortical sensory responses (Sörqvist et al., 2012). Some researchers propose that engaging working memory resources via a secondary task likely affects the ability of attention to act as a gatekeeper for early sensory inputs (Sörqvist et al., 2012). Accordingly, evidence shows that cognitive load interferes with attention processes (de Fockert et al., 2001; Lavie, 2010; Lavie et al., 2004), multisensory integration and postural control (Andersson et al., 1998; Redfern et al., 2001), and proprioceptive ability matching performance (Goble et al., 2012), possibly by interfering with early and late perceptual and attentional processes (Lavie, 2010). Note that these processes are likely involved in the RHI.

Following the idea that working memory also contributes to binding information together to form of a unified percept (Quak et al., 2015), we reasoned that engaging working memory resources away from the RHI would impair inferential processes involved in generating the illusion. Consistent with our hypothesis, evidence shows that attentional load weakens the McGurk effect (Alsius et al., 2005, 2007, 2014; Buchan & Munhall, 2011a, 2012). However, in contrast to this prediction, a recent study showed that modulating working memory capacities via a load manipulation hardly reduces the strength of the RHI, thereby providing support to the notion that this illusion is relatively automatic and requires minimal cognitive effort (Fahey et al., 2018). Our research therefore provides the means to replicate and validate this null outcome.

Our second manipulation aimed to assess whether instructions intended to shift one’s focus to emphasise somatosensory inputs or the visual ones would modulate the strength of the illusion. This strategy follows from the idea that multisensory integration likely involves a weighting procedure that fuse sensory signals together, while attention critically change these weights (Kayser & Shams, 2015; Talsma et al., 2010). Based on this construal, we hypothesised that overweighting tactile or visual inputs by attending to one versus the other would alter the emergence of the RHI. In other words, emphasising signals pertaining to the real arm, as opposed to a fake one, would better ground the experience of body ownership into somatosensations, thus reducing the influence of seeing the fake arm being stroked. In this way, attending tactile sensations could increase awareness of the arm’s true position in space and afford better detection of perceptual mismatches between seen and felt stroking, and therefore impede the emergence of distortions.

This experimental strategy is consistent with mounting evidence showing that task instructions can influence perception (cf. Brass & de Houwer, 2017). For example, attentional instructions influence the dynamics of perception in the context of binocular rivalry and ambiguous figures (Liebert & Burk, 1985; Paffen & Alais, 2011; Toppino, 2003). Similarly, one sensory modality can dominate the others and yield cross-model biases (Cao et al., 2019). A similar effect can be observed in the context of body ownership, where sight can exert great influence over proprioception in a manner that changes how tactile sensations are interpreted and ascribed to body representations (Botvinick & Cohen, 1998; Carey et al., 2019; Chancel et al., 2021; Hagura et al., 2007; Holmes et al., 2006; Maselli & Slater, 2013; Pavani et al., 2000; Ponzo et al., 2018; Willis et al., 2021; but see Guterstam et al., 2013). The assumption that researchers can alter this preferential treatment is therefore central to the present work.

Ample evidence highlights the centrality of attention in multisensory integration (Fernández et al., 2015; Koelewijn et al., 2010; Mozolic, Hugenschmidt, et al., 2008; Mozolic, Joyner, et al., 2008; Senkowski et al., 2005; Talsma et al., 2007, 2010; Talsma & Woldorff, 2005). Attending to a specific modality typically tunes down the processing of other modalities; whereby, unattended sensory signals hardly influence perception (Knudsen, 2007; Laurienti et al., 2002; Mozolic, Hugenschmidt, et al., 2008; Mozolic, Joyner, et al., 2008). As a case in point, providing instructions to attend to different sensory information interferes with the otherwise automatic sensory predominance of vision during the McGurk effect (Buchan & Munhall, 2011b). Likewise, suggestions to prioritise auditory input leads to a weaker McGurk illusion in highly hypnotisable individuals (Déry et al., 2014; Lifshitz et al., 2013).

Consistent with these findings, previous work alludes to the direct role of attentional factors in the RHI (Tsakiris, 2017). For instance, individuals showing higher interoceptive sensitivity—a predisposition to attend to, perceive, and report internal bodily signals such as heartbeats accurately—typically experience a weaker RHI (Schauder et al., 2015; Tsakiris et al., 2011; but see Horváth et al., 2020).^2^ Note that although researchers have historically employed the term interoception to describe “visceroception” (internal perception of viscera—heart, gut, lungs, etc.), the term is increasingly conceptualised more broadly as to also include proprioception and skin sensations (Björnsdotter et al., 2010; Cameron, 2001, 2002; Ceunen et al., 2016; Craig, 2002; von Mohr & Fotopoulou, 2019). Furthermore, the sense of touch serves as an auxiliary proprioceptive cue (Blanchard et al., 2011; Moscatelli et al., 2016, 2019). Therefore, in the present work, rather than operationalising interoception as a trait or ability, we attempted to manipulate it via explicit instructions. We instructed participants to either focus their attention on the tactile sensation of their real hand or on external visual information (i.e., the sight of the rubber hand; “exteroception”). Given that interoceptive and exteroceptive cues appear antagonistic (Tsakiris, 2017), this experimental procedure should modulate the illusion such that participants will report a stronger illusion with visual instructions, which would prioritise the exteroceptive information.

## Method

### Participants

We used convenience sampling and recruited 38 undergraduate students through the psychology participant pool system at McGill University—data collection stopped at the end of the semester. Each participant gave informed consent and received two credits for their participation. We excluded five participants due to requests from participants to stop the experiment, excessive knowledge about the goals of the experiment, or experimenter error. For purposes of analysis, we kept data from 33 participants (*M*_age_ = 20.6 years, *SD*_age_ = 1.4 years, 70% females, 79% right-handed, 58% White, 24% Asian, 6% Other; demographic data was lost for four participants). We provide the factor score data and analyses on the Open Science Framework (https://osf.io/qc2hm/). The Research Ethics Board at McGill University approved this study prior to data collection.

### Materials

The fake hand consists of a realistic silicon anatomical prosthesis including the right hand, forearm, arm, and shoulder (produced by Milsuite FX Inc.). A self-made separator wrapped with a silver-pink cloth occluded the real right hand of participants from their view. Participants 1 to 13 indicated their responses on a paper questionnaire displaying eight-item visual analogues ranging from 0 (“*I do not agree at all*”) to 7 (“*I agree completely*”) following statements such as, “I felt as if the hand I saw was my hand.” Participants 14 to 33 instead indicated their responses on the computer—to reduce paper consumption—by typing a number from 0 to 7 in a similar fashion, but without a visual analogue. In brief, the questionnaire assesses changes in phenomenological experience as a function of our experimental conditions. To design it, we adapted 35 questions from other researchers adopting the RHI methodology (Farmer et al., 2012; Gonzalez-Franco et al., 2014; Guterstam et al., 2011; Longo et al., 2008; Rohde et al., 2011; Tsakiris et al., 2011).

### Procedure

**Figure 1.**
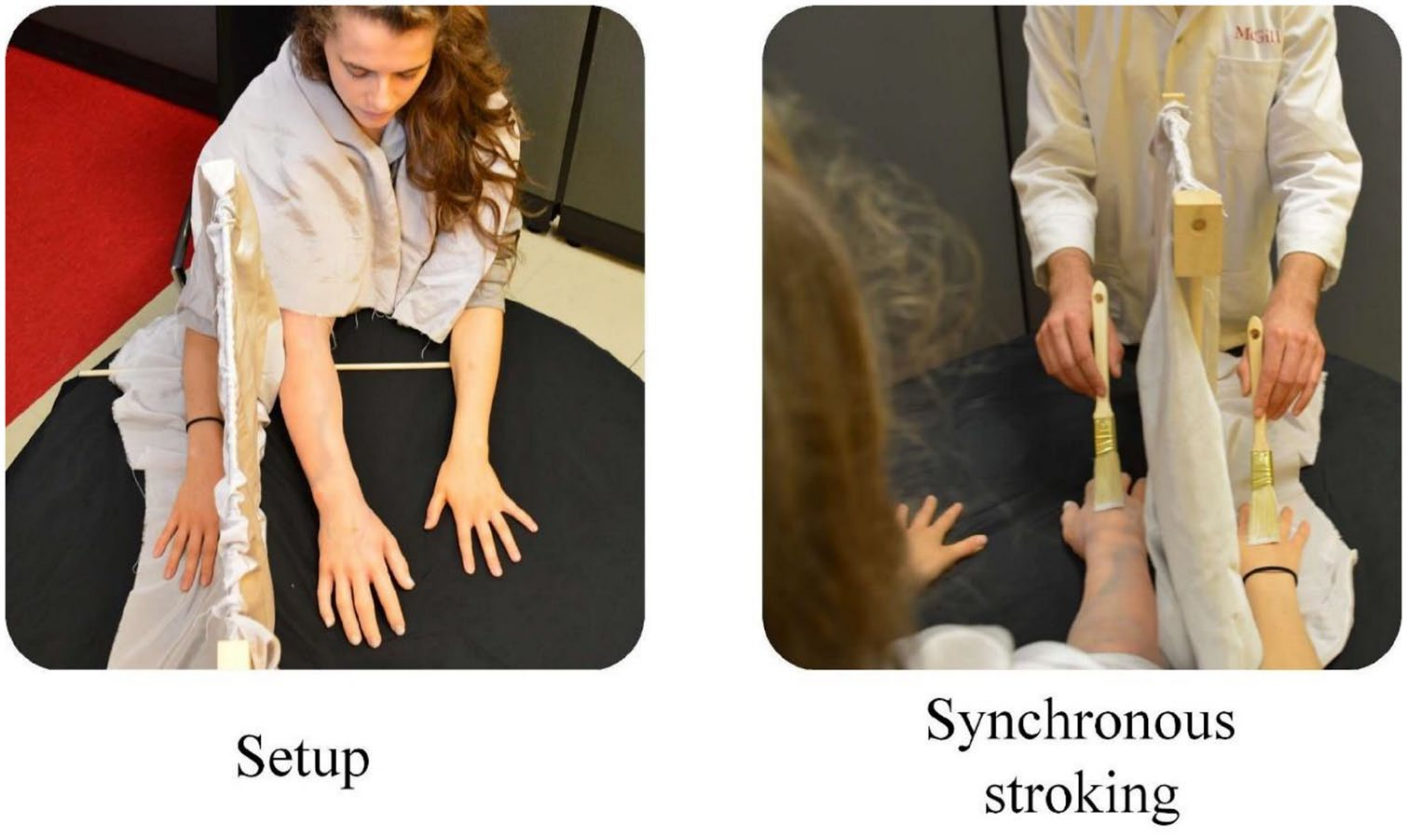
Rubber hand illusion setup. Left: experimental setup with fake silicon arm between the two real hands and the occluder. Right: participant view during synchronous stroking.

The experimenter explained the general goals of the study to participants as “examining the cognitive dimensions of body ownership illusions” and that the experiment aimed to “explore body sensations and body perceptions.” Next, they signed a consent form prior to the experiment. We noted down participant demographics, after which participants entered the testing room. Participants then sat on a chair in front of a table supporting the experimental apparatus, and we positioned their right arm on the table on the right of an occluding partition so that their hand and arm were out of sight. They similarly positioned their left hand on the table, but in a clearly visible fashion in their left field of view. We then placed a fake silicon arm on the left of the occluder so that the shoulder section of the fake arm leaned against the frontal part of the right shoulder of participants. The angle of the fake arm differed from the natural position of the real arm by about 5 cm—the width of the partition. We then put a sheet to cover their shoulders to visually mask the distinction between their real arm and the fake arm. After giving proper instructions about the procedure, we synchronously stimulated both the real and fake arms with small paintbrushes for approximately 2 min to induce the illusion (Figure 1).

Participants underwent the illusion across four counter-balanced experimental conditions: (a) cognitive load and attend visual signal, (b) no load and attend visual signal, (c) cognitive load and attend tactile sensation, and (d) no load and attend tactile sensation.^3^ During cognitive load, the experimenter exposed participants to a string of characters composed of six random digits and letters (e.g., A7D3X2) that appeared for approximately 1 s on a computer screen. Participants had to remember this character string until after the stimulation was over, after which they had to spell out the character string they had received (i.e., about 2 min).^4^ In the no load condition, participants merely had to remember a string of characters composed of the same random number or letter (e.g., EEEEEE). Regarding the attended sensory signal manipulation, we instructed participants to attend to their visual perception of the stroking of the hand in front of them in the visual signal condition:

Please attend to the sight of the hand in front of you while it is being stroke until further instructions. Please do not attend to your real hand or to its tactile sensations. Attend to the hand you see.

In the tactile signal condition, we instructed participants to focus on the tactile sensation of the paintbrush touching their real hand while keeping their gaze focused on the fake hand:

Please attend to the tactile sensation of your real hand while it is being stroke until further instructions. Continue to gaze at the fake hand but do not focus on it. Attend to the sense of touch on your real hand.

After the stimulation, participants filled out a self-administered questionnaire concerning their experience for each of the four conditions, so that each participant filled out four questionnaires in total. This questionnaire assessed various dimensions relating to the phenomenology associated with the RHI. At the end, we debriefed each participant. In total, the experiment took approximately 45 min.

### Factor analysis

In the current study, we first carry out a factor analysis rather than using the pre-established dimensions of body ownership identified by Longo et al. (2008). Several reasons motivate this approach. First, independent researchers have yet to validate the questionnaire of Longo et al. (2008), providing an opportunity to corroborate the proposed structure of body ownership in a different sample. Second, there were known limitations to the Longo et al. (2008) methodology (cf. Fabrigar et al., 1999). For instance, Longo et al. (2008) used Principal Component Analysis (PCA), while it would have been more appropriate to use Exploratory Factor Analysis (EFA) due to the implicit goal of generalising the findings to the population level rather than restraining the interpretation to the sample level (Fabrigar et al., 1999; Field et al., 2012). Similarly, given the intercorrelation between the factors, the factor rotation should have been oblique, as opposed to orthogonal (Fabrigar et al., 1999; Field et al., 2012).

Therefore, carrying out our own factor analyses improves the validity of the psychometric dimensions used in this study. We also include additional items used in more recent RHI studies that are not part of the questionnaire developed by Longo et al. (2008), making it that much more critical to conduct new factor analyses and therefore validate the relevant constructs. We note however that given our use of a different experimental design and additional items, this factor analysis serves as a rather limited and context-specific “validation” of Longo and colleagues (2008). Readers should appraise the theoretical value of this analysis accordingly.

Following the work of Longo et al. (2008), we first attempted to replicate their psychometric findings using an identical analytical procedure: a PCA with orthogonal varimax rotation.^5^ Here, we pooled data from our repeated measures design. This strategy aims to increase the number of data points to benefit the identification of factors (Schopflocher & Ulrich, 2005). Thus, we treated each data point separately—a procedure that yielded 132 observations in total. Obviously, this approach voids independence between data points and assumes that we should recover the same latent structure across all conditions. However, the idea that our top-down factors would dramatically change the phenomenology of the RHI such that it would modify the latent structure seems rather implausible. Furthermore, based on Monte Carlo simulations, between 60 and 100 observations are adequate for high items communalities values (⩾.6), whereas with lower communalities (~.5), adequate samples ought to contain between 100 and 200 observations (de Winter et al., 2009; Fabrigar et al., 1999; MacCallum et al., 1999, 2001; Mundfrom et al., 2005; Russell, 2002). In turn, these predictions hold when the ratio of items to factors is medium to high (3.3–6.7), though the higher the ratio the better. Given our 35-item average communalities of .60 and our high item factor ratio of 8.75, 132 observations therefore seem quite adequate.

Our PCA results for the most part replicated those of Longo et al. (2008). We thus followed up with a Confirmatory Factor Analysis (CFA) to validate the latent structure of the questionnaire developed by Longo et al. (2008). We accordingly hypothesised a four-factor solution and excluded items that loaded less than .5 or that were not included in the original analysis. The CFA comprised 21 of the questions from Longo et al. (2008) and assumed four factors based on their four-factor model for synchronous stroking only, namely, embodiment of the rubber hand, loss of own hand, movement, and affect. We specified the model as follows: embodiment → Items 1–8, 15–16; loss of hand → Items 14, 19, 28–30; movement → Items 12, 17, 34; and affect → Items 20–22 (average communalities of these 21 items = .68; item:factor ratio = 5.25).

However, the model poorly fitted the data (but see van Prooijen & van der Kloot, 2001, for a discussion on the relationship between CFA fit and factor structures obtained through factor analysis). We therefore opted to follow up with an EFA to clarify the underlying structure from our data set. We performed EFA using the Minimal Residual method to extract the factors, and again we used an oblique (i.e., “oblimin”) rotation due to the intercorrelation between our factors (Fabrigar et al., 1999; Field et al., 2012). We obtained the weighted standardised factor scores using Bartlett’s method (as suggested by DiStefano et al., 2009, for oblique rotation). These analyses were completed in R version 3.4.2 (R Core Team, 2021) using packages nFactors (for the scree plot; Raiche & Magis, 2020), psych (for PCA and EFA; Revelle, 2018) lavaan (for CFA; Rosseel, 2012), and effsize (for effect sizes; Torchiano, 2020).

### Experimental analyses

Using the weighted standardised factor scores obtained from the EFA, we then used hierarchical linear regression models to determine whether the four factors identified by the EFA—that is, *embodiment of rubber hand*, *loss of own hand*, *feeling of having two right hands*, and *affect*—varied as a function of instructions and cognitive load (Gelman & Hill, 2006). This analytical approach proves to be well-adjusted for repeated measures designs, to the point of outperforming the classic analysis of variance (ANOVA) model (Boisgontier & Cheval, 2016; Quené & van den Bergh, 2004). Within our framework, the *embodiment* component refers to feelings of ownership and control over the fake hand; the *loss of own hand* component indexes loss of control and feelings of numbness over the real hand; the *feeling of having two right hands* component corresponds to the impression of feeling both the rubber hand and the real hand simultaneously; and finally, the *affective* dimension follows from questions pertaining to the pleasantness of the experience. Here, instructions (i.e., visual vs. tactile) and cognitive load (i.e., no load vs. load) were included in a stepwise fashion as fixed factors, while participants were included as random factors. We selected the best fitting model based on a likelihood-ratio chi-square test and the Bayes Information Criterion (BIC). Moreover, we relied on Bayes Factors to evaluate how evidence weights in favour of the null hypothesis versus the alternative one (Wagenmakers, 2007). We estimated Bayes Factors via the BIC using the following equation: BF_01_ = e^ΔBIC_10_/2^. We fitted the hierarchical linear regression models using the MATLAB (MathWorks inc., Version R2020a) fitglme function.

## Results

### PCA

In total, 11 of the 27 items used by Longo et al. (2008) did not load on the same factor or also loaded on an additional factor in our PCA results (our Items 1, 17–19, 21, 23–26, 32, and 34; Longo’s Items 11, 14, 15, 17, 18, 21, and 23–26). See Table S1 for complete loadings and comparison with Longo et al. (2008).

### CFA

The data did not meet the assumption of multivariate normality for CFA (i.e., the variables were not normally distributed). We accordingly used a robust maximum likelihood estimator with Huber-White standard errors and a scaled test statistic (asymptotically equal to the Yuan-Bentler test statistic). The results of the CFA are available in Table 1. Ultimately, none of the indices meet the commonly accepted minimum criteria and revealed poor fit of the data (Schreiber et al., 2006).

**Table 1.** Confirmatory factor analysis results.

	χ^2^	*df*	χ^2^/*df*	*p*	CFI	TLI	RMSEA	SRMR
Reference value^a^	Ratio of χ^2^ to *df* < 2 or 3			>.05	⩾.95	⩾.95	<.06–.08	⩽.08
Current study^b^	600.08	183	3.28	<.001	.79	.76	.13	.11

CFI: comparative fit index; TLI: Tucker–Lewis index; RMSEA: root mean square error of approximation; SRMR: standardised root mean square residual.

a As proposed by Schreiber et al. (2006).

b Excludes items not used by Longo et al. (2008) and those that loaded less than .5 in their study. It includes 21 of the questions from Longo et al. (2008) and four factors based on their four-factor model (for synchronous stroking): (a) embodiment of rubber hand, (b) loss of own hand, (c) movement, and (d) affect.

### EFA

Following the poor fit of the CFA, we opted for an EFA. We verified the sampling adequacy of the individual items with the Kaiser-Meyer-Olkin (KMO) measure. We removed three items with KMO values smaller than .7 (considered mediocre by Kaiser, 1974): Items 22, 27, and 35. All other values were greater or equal to .7. We additionally removed three items with fewer than five correlations greater than .3 (Items 9, 31, and 33). Obtaining a determinant of the correlation matrix greater than 1e-5 (a common rule of thumb, Field et al., 2012) would have required us to drop an additional 13 items with correlations greater than .7 (Items no 1–8, 14, 16, 18, 19, and 30). We deemed this solution impracticable given that such a high attrition would represent close to 50% of our questions. Instead, we opted to simply drop one extra item: Question 4 because its wording was virtually identical to Item 2 and correlated with it at .9 (indicating the question may have been redundant). Overall, this resulted in the exclusion of seven items (4, 9, 22, 27, 31, 33, and 35), leaving us with 28 items (average communalities of these 28 items = .65; item:factor ratio = 7). This procedure leaves us with a suboptimal correlation matrix determinant of 6.87e-12, though the overall KMO measure of sampling adequacy was a hair more than .9—a reliable score (Kaiser, 1974). However, this solution entails that the high multicollinearity of the data set represents a limitation of the current factor analysis because it entails that most items load onto the same construct. Bartlett’s test of sphericity, *x*^2^(378) = 3,105.945, *p* < .001, confirmed the inter-item correlations were large enough for the analysis. Five components had eigenvalues more than Kaiser’s criterion of 1 and together explained 70.92% of the variance, though the point of inflexion on a scree plot justified keeping three components that explained 62.37% of the variance. We decided to retain four components explaining 66.70% of the variance in light of both Kaiser’s criterion and the scree plot, as well as of previous findings by Longo et al. (2008).

**Figure 2.**
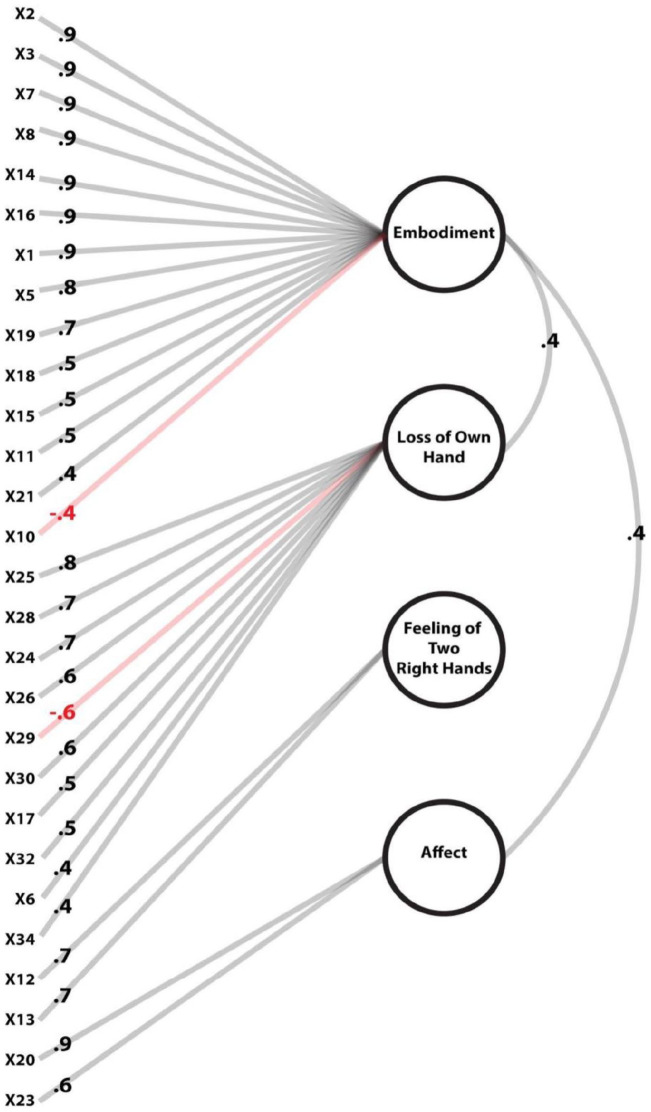
Exploratory Factor Analysis. Numbers on the left represent item numbers; numbers on the lines represent item loadings on their primary factor or intercorrelation between factors. The red lines represent negative loadings.

**Figure 3.**
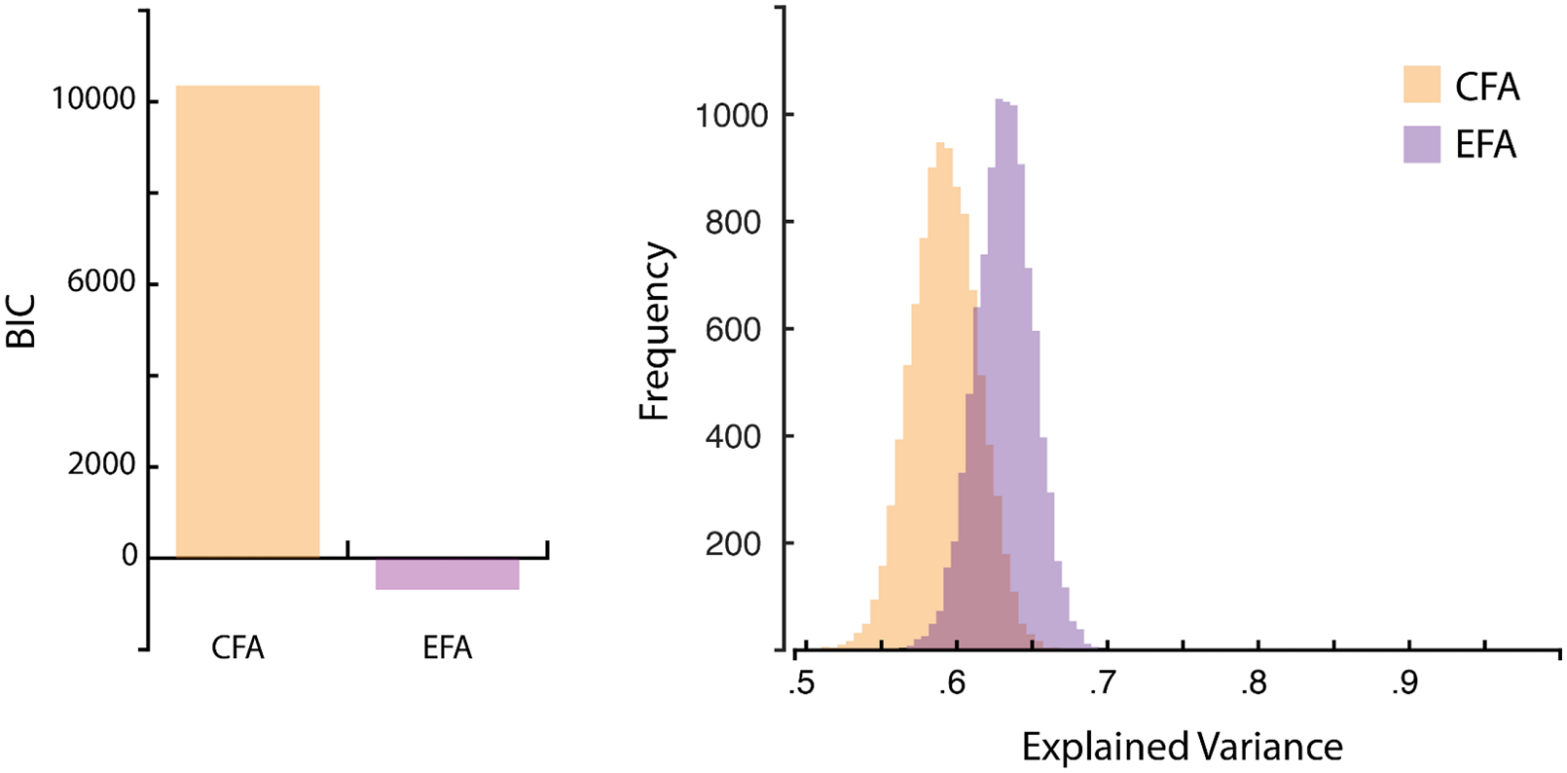
Model comparison: confirmatory versus exploratory factor. Left panel: the large BIC difference suggests a much better fit for Exploratory Factor Analysis (EFA) than Confirmatory Factor Analysis (CFA). Right panel: distributions of bootstrapped (10,000 samples each) total variances explained by the CFA and EFA models, respectively. Note that for the CFA, 250 bootstrapped variances explained (out of 10,000) were greater than one due to the bootstrapping process, so were excluded for this figure. The overlapping distributions suggest both the CFA and EFA models explain comparable total variances, with the EFA explaining marginally more. BIC: Bayes Information Criterion.

A four-factor solution revealed that although residuals distributed normally, more than 50% of residuals (85.71%) were larger than .05, and that the root-mean-square residual (.21) was more than .08, putting these values above the commonly accepted limits. Field et al. (2012) suggests that such results encourage extracting more factors; however, extracting more factors only worsened both issues, and extracting fewer factors did little to help reach the acceptable values. This issue likely reflects our low sample size. Figure 2 graphically displays the model. Figure 3 displays how the EFA model provided a much better BIC fit (–691.09) than the CFA model (10,351.36), while also explaining marginally more variance (based on 10,000 bootstraps per model). The EFA scale overall had high internal consistency (Cronbach’s α = .95, 95% confidence interval [CI] = [0.94, 0.96]).

The table of loadings/pattern matrix (Table 2) suggests the four components represent (a) embodiment of rubber hand, (b) loss of own hand, (c) feeling of having two right hands, and (d) affect. See the Supplemental Material, Table S2 for the equivalent table of loadings without excluded items (for those interested in where those would have loaded), Table S3 for the table of correlations, and Table S4 for the structure matrix. The Cronbach’s alphas for the four factors were as follows: embodiment of rubber hand (.95, 95% CI = [0.94, 0.97]), loss of own hand (.88, 95% CI = [0.85, 0.91]), feeling of having two right hands (.72, 95% CI = [0.63, 0.81]), and affect (.67, 95% CI = [0.56, 0.78]).

**Table 2.** Factor loadings/pattern matrix (Items 4, 9, 22, 27, 31, 33, and 35 excluded) for Exploratory Factor Analysis (EFA) with oblique (“oblimin”) rotation and four factors.

Item no	During the block . . .	Expected dimension	Embodiment of rubber hand	Loss of own hand	Two right hands	Affect	Communalities
1	I felt the touch of the brush on the hand I saw.	Ownership^a^	0.853				0.818
2	I felt as if the hand I saw was my hand.	Ownership^b^	0.931				0.852
3	It seemed like the hand I saw was part of my body.	Embodiment of rubber hand^c^ (Ownership^d^)	0.907				0.84
5	It seemed like the hand I saw belonged to me.	Embodiment of rubber hand^c^ (Ownership^d^)	0.821				0.767
6	It seemed like the hand I saw began to resemble my real hand.	Embodiment of rubber hand^c^ (Ownership^d^)					0.525
7	It seemed like I could have moved the hand I saw if I had wanted.	Loss of own hand^c^	0.893				0.775
8	It seemed like I was in control of the hand I saw.	Embodiment of rubber hand^c^	0.877				0.756
10	I felt the touch of the brush on my (real) hand.	Disownership of the real hand^a^					0.391
11	It no longer felt like my (real) hand belonged to my body.	Disownership of the real hand^a^	0.509	0.531			0.61
12	It felt as if I had two right hands.	Feeling of having two right hands^a^			0.673		0.557
13	I felt the touch of the brush on both hands at the same time.	Feeling of having two right hands^a^			0.667		0.628
14	It seemed like the touch I felt was caused by the brush touching the hand I saw.	Embodiment of rubber hand^c^	0.857				0.784
15	It seemed like the hand I saw was in the location where my hand was.	Embodiment of rubber hand^c^ (Location^d^)	0.523				0.541
16	It seemed like my hand was in the location where the hand I saw was.	Embodiment of rubber hand^c^ (Location^d^)	0.855				0.798
17	I felt as if my (real) hand were drifting towards the left (towards the fake hand).	Movement^c,e^		0.528			0.432
18	It seemed like I couldn’t really tell where my (real) hand was.	Loss of own hand^c^	0.546				0.633
19	It seemed like my (real) hand had disappeared.	Loss of own hand^c^	0.732				0.79
20	I found the experience enjoyable.	Affect^c^				0.857	0.819
21	I found the experience interesting.	Affect^c^					0.306
23	I found myself liking the hand I saw.	No loading^c^				0.567	0.566
24	I had the sensation of pins and needles in my hand.	Deafference^c^ (asynchronous)		0.701			0.468
25	I had the sensation that my hand was numb.	Deafference^c^ (asynchronous)		0.784			0.625
26	It seemed like the experience of my hands was less vivid than normal.	Deafference^c^ (asynchronous)		0.635			0.519
28	It seemed like I was unable to move my hand.	Loss of own hand^c^		0.71			0.631
29	It seemed like I could have moved my hand if I had wanted.	Loss of own hand^c^		−0.633			0.474
30	It seemed like my hand was out of my control.	Embodiment of rubber hand^c^		0.613			0.54
32	It felt as if my (real) hand were turning “rubbery.”	Control statement^a,c,e^					0.486
34	It appeared (visually) as if the fake hand was drifting to the right (towards my real hand).	Control statement^a,e^, movement^c^					0.242
	Eigenvalues		8.826	5.021	1.757	1.568	
	Percentage variance explained		31.5	17.9	6.3	5.6	

Component loadings less than 0.5 are not displayed. Items were adapted from the following: ^a^Guterstam et al. (2011), ^b^Gonzalez-Franco et al. (2014), ^c^Longo et al. (2008), ^d^Tsakiris et al. (2011), and ^e^Rohde et al. (2011).

#### Embodiment of rubber hand

“Embodiment of rubber hand” emerged as expected, encompassing most items from previous studies, except that according to the PCA by Longo et al. (2008), Items 7, 18, and 19 should have loaded on the “Loss of own hand” factor, and Item 11 should have loaded on “Loss of own hand” as well according to theorisation by Guterstam et al. (2011).

#### Loss of own hand

“Loss of own hand” emerged as expected, including many items from previous studies, except that according to the PCA by Longo et al. (2008), Item 30 should have loaded on the “Embodiment” factor, Item 17 should have loaded on the “Movement” factor, and Items 24–26 should not have loaded anywhere (as in their study it only loaded on a “Deafference” component that only emerged in asynchronous stroking conditions).

#### Feeling of having two right hands

The structure emerging from this third factor does not seem to reflect the third factor identified by Longo et al. (2008), “Movement.” In fact, Items 12 and 13 were not used by Longo, but according to Guterstam et al. (2011), these two questions should belong to a dimension they named “Feeling of having two right hands.” It seems like in the current experiment, this dimension replaced the “Movement” dimension. Only two of our items previously loaded on the “Movement” dimension in Longo’s research (Items 17 and 34). It seems that Item 17 now loads on the “Loss of own hand” dimension, associating the drift to losing own’s hand. Note that we did not include Longo’s question: “. . . it seemed like I had three hands,” which previously loaded on “Movement” as well because we thought it was similar enough to our Item 12 (feeling like having two right hands more or less implies feeling three hands in total). Furthermore, Item 34 previously loaded on “Movement” as well, whereas here it did not load anywhere, perhaps because we used the modified wording by Guterstam et al. (2011), which added the specification that the rubber hand was “visually” drifting towards the real hand.

#### Affect

Although we had to remove Item 22, the “Affect” factor emerged as expected, with the difference that Item 23 had previously not loaded on any factor in the PCA by Longo et al. (2008).

#### Items with no loadings greater or equal to .5

According to the PCA by Longo et al. (2008), Item 6 should have loaded on the “Embodiment” factor. Item 10 may have been expected to load on “Loss of own hand” in light of the theorisation by Guterstam et al. (2011). In the study by Longo et al. (2008), Item 21 loaded on the “Affect” dimension. Item 34 may have also been expected to load on the “Movement” factor according to them; however, we used a modified wording version (Guterstam et al., 2011; Rohde et al., 2011) that was classified as a control statement (perhaps because of the emphasis on a “visual” drift).

### Experimental condition

**Figure 4.**
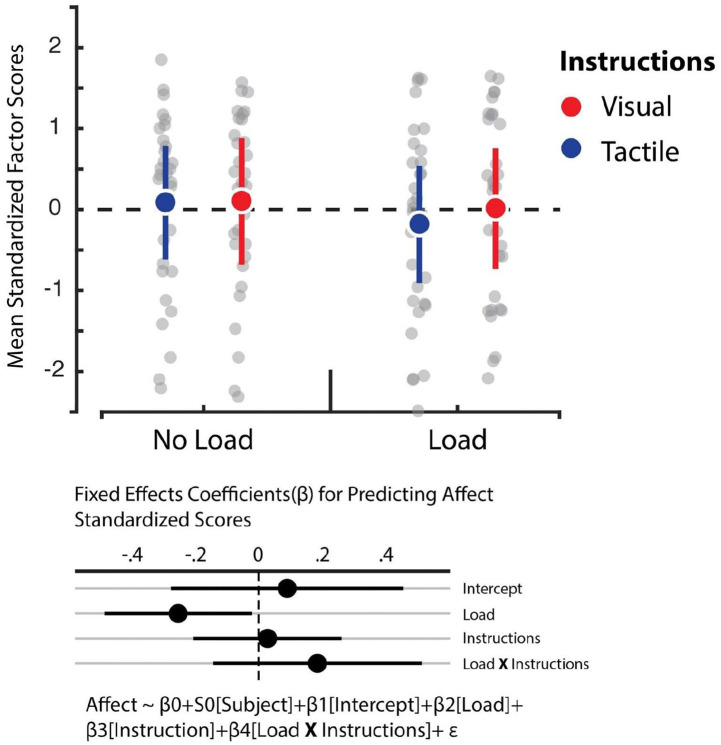
Embodiment factor. Averaged standardised factor scores for the “Embodiment of rubber hand” dimension (y-axis). In the original scale, participants could choose between 0 (“*I do not agree at all*”) and 7 (“*I agree completely*”). Regression analyses revealed that Instructions were a statistically reliable predictor of embodiment, (β = 0.5, *SE* = 0.1, 95% CI = [0.29, 0.7]. Error bars represent 95% bootstrapped confidence intervals.

**Figure 5.**
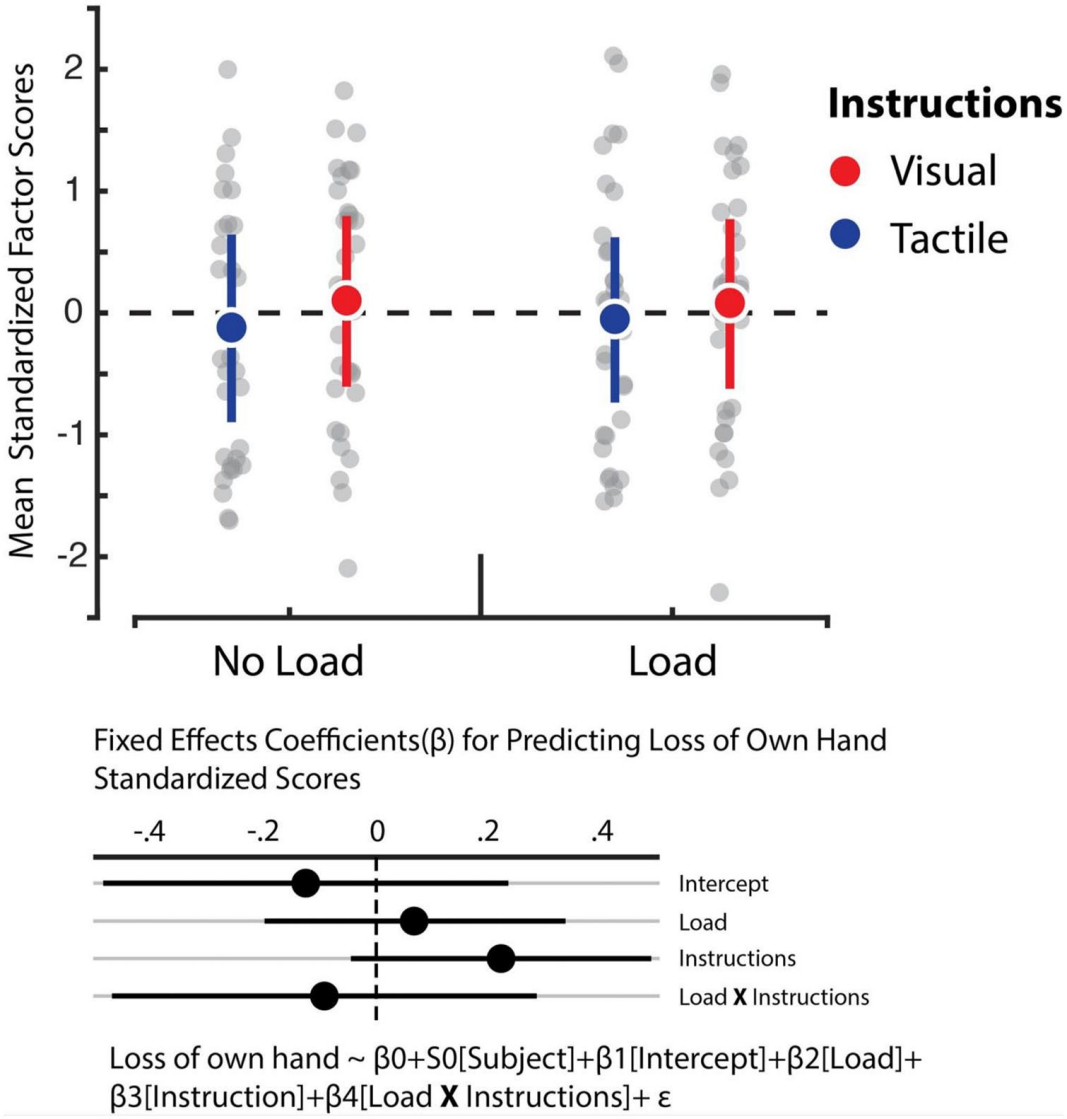
Affect factor. Averaged standardised factor scores for the “Affect” dimension (y-axis). In the original scale, participants could choose between 0 (“*I do not agree at all*”) and 7 (“*I agree completely*”). Regression analyses revealed that load was a statistically reliable predictor of affect (β = –0.17, *SE* = 0.09, 95% CI = [–0.35, –0.009]). Error bars represent 95% bootstrapped confidence intervals.

**Figure 6.**
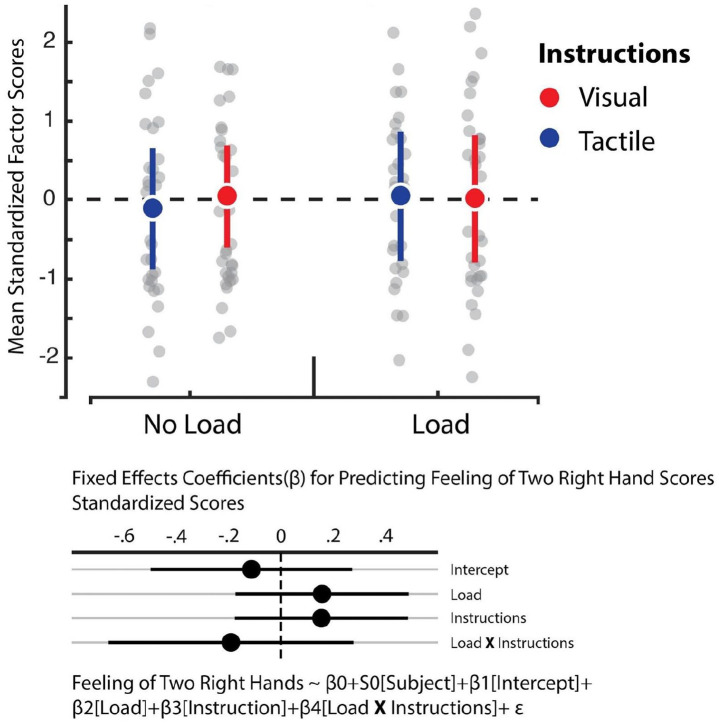
Loss of one’s own hand factor. Averaged standardised factor scores for the “Loss of own hand” dimension (y-axis). In the original scale, participants could choose between 0 (“ *I do not agree at all* ”) and 7 (“*I agree completely*”). There were no significant effects. Error bars represent 95% bootstrapped confidence intervals.

**Figure 7.**
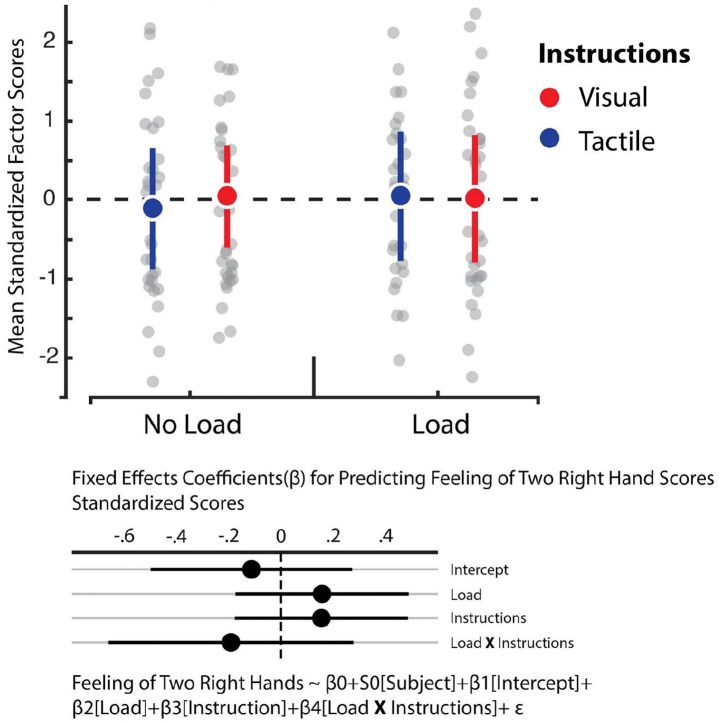
Feeling of having two right hands factor. Averaged standardised factor scores for the “Feeling of having two right hands” dimension (y-axis). In the original scale, participants could choose between 0 (“*I do not agree at all*”) and 7 (“*I agree completely*”). There were no significant effects. Error bars represent 95% bootstrapped confidence intervals.

We relied on hierarchical linear regression models to determine whether the instructions and load manipulations influenced feelings of embodiment, increased feelings of having two right hands, affective components of the RHI, and reduced feelings of one’s own hand (see Figures 4 to 7). The best fitting model for predicting feelings of embodiment revealed that instructions was the sole statistically reliable predictor (β = 0.5, *SE* = 0.1, 95% CI = [0.29, 0.7]; see Tables S5 and S6). For comparison, the Cohen’s *d* for the difference between visual and tactile instructions revealed a medium effect size of 0.51. This outcome indicates that tactile instructions decrease feelings of embodiment in the RHI relative to visual ones. In turn, however, Bayes factor analysis comparing the baseline model against the alternative model, which comprised only the load variable, revealed that evidence weighted in favour of the null hypothesis (BF_01_ = 11.48). Corroborating previous work, our data indicate that the load manipulation did not influence feelings of embodiment (Fahey et al., 2018). Furthermore, we observed no influence of instructions and load on the feeling of having two right hands and loss of feeling towards one’s own hand. In both cases, the data were best fitted by the baseline model which solely comprised the intercept (see Tables S7 and S8). Bayes factor confirmed this assessment whereby evidence largely supported the null model for the feeling of having two right hands against the load (BF_01_ = 9.9) and instruction (BF_01_ = 10.02) manipulations. Likewise, evidence also supported the null model for losing feeling in one’s own hand with respect to the load manipulation (BF_01_ = 11.13), though evidence was ambiguous regarding the instruction manipulation (BF_01_ = 2.11). Finally, we observed that the best fitting model for predicting the affective component solely included load as a reliable predictor (β = –0.17, *SE* = 0.09, 95% CI = [–0.35, –0.009]; see Tables S9 and S10). However, the Cohen’s *d* for the difference between load and no load indicated a small effect size of 0.16. This outcome shows that cognitive load reduces this dimension of the RHI, albeit weakly.

## Discussion

The present study aimed to explore the influence of top-down components, such as attention and working memory, on the RHI. To this end, we investigated whether the illusion is vulnerable to the availability of working memory resources by manipulating a cognitive load, and by varying task instructions with regards to the attended sensory event. Our approach followed previous work that highlighted various phenomenological dimensions of the RHI (Longo et al., 2008). In this regard, the CFA derived from this work was suboptimal, while the EFA provided better fit of the data. This improvement likely follows from the additional items we used in our research. We predicted that reducing the availability of cognitive resources and instructing participants to attend to somatosensory sensations would lessen the magnitude of the RHI. Our results partly support these predictions as task instructions modulated feelings of embodiment, wherein individuals reported lower feelings of embodiment towards the fake arm when they focused on tactile as opposed to visual sensations. In this regard, the experience of embodiment is broad and encompasses several subcomponents, such as feelings of ownership and control over the fake hand, or impressions that the felt touch emerges from the fake hand (i.e., referral of touch; de Vignemont, 2011). Hence, shifting attention to different sensory inputs shapes the phenomenology of body ownership. Conversely, our cognitive load manipulation solely affected the pleasantness of the experience—a somewhat unexpected outcome that likely highlights the unpleasantness of cognitive effort. In this regard, our findings replicated previous work in showing the RHI hardly relies on top-down cognitive resources (Fahey et al., 2018). Furthermore, our variables did not influence other dimensions, namely, feelings of having two right hands and of losing one’s own hand. Null findings are notoriously difficult to interpret (e.g., Wagenmakers, 2007). Here, we resorted to a Bayesian approach to evaluate whether evidence supports the null hypothesis (Dienes, 2014). With the exception of feelings of losing one’s own hand where evidence was inconclusive, Bayes factors supported the null hypothesis in all cases. In sum, these results contribute to a growing body of evidence suggesting that various higher-order cognitive processes, including attention, can modulate the RHI (Dempsey-Jones & Kritikos, 2014; Kilteni et al., 2015).

### Exploring the effects of task instructions

Our findings emphasise the influence of task instructions and attention in shaping the phenomenology of the e*mbodiment of rubber hand* dimension. We speculate that these modulations reflect the putative central role of visual processing in bodily self-consciousness (Deroy et al., 2016; Faivre et al., 2015).^6^ Prevailing views argue that the combination of visual and tactile inputs overwrites prior proprioceptive knowledge, thereby altering body representations during the RHI (Botvinick & Cohen, 1998; Ehrsson et al., 2004; Hagura et al., 2007; Pavani et al., 2000). The current work expands this viewpoint by showing how focusing attention to visual inputs heightens feelings of embodiment in the context of the RHI compared to when individuals instead focus on somatosensations. This outcome intimates that the emergence of such feelings follows from the attentional prioritisation of visual information towards the prosthesis, thereby facilitating its integration within existing body representations (though we note illusions of body ownership can ultimately arise without the contribution of vision, e.g., in the somatic version of the RHI, Ehrsson et al., 2005; also see Ehrsson, 2020, for a review).

This interpretation aligns with previous viewpoints (Botvinick & Cohen, 1998; Carey et al., 2019; Chancel et al., 2021; Hagura et al., 2007; Maselli & Slater, 2013; Pavani et al., 2000; Ponzo et al., 2018). Conversely, focusing on tactile information seemingly grounds prior body representations—for example, by allowing participants to better notice discrepancies between seen and felt touch—therefore impeding the ability of visual inputs to induce further alterations. Indeed, the sense of touch can act as an auxiliary proprioceptive cue (Blanchard et al., 2011; Moscatelli et al., 2016, 2019).

These results dovetail previous findings showing that greater awareness of internal bodily signals (“interoception”) similarly weakens the RHI (Schauder et al., 2015; Tsakiris et al., 2011). By improving awareness of the true body position (through increased attention to tactile inputs), enhanced somatosensory processing may have consolidated a firmer and clearer body representation–that is, less prone to distortions–thus leading to attenuated feelings of embodiment of the fake hand. Our findings therefore highlight how the interplay between visual and tactile inputs, mediated through attention processes, shapes a core phenomenological dimension of body ownership.

Hence, in the context of multisensory processing, attention seemingly influences the RHI by biasing the competition between sensory signals, which impairs the processing of the unattended signal—a phenomenon known as cross-modal *deactivation* (Fernández et al., 2015; Knudsen, 2007; Laurienti et al., 2002; Mozolic, Hugenschmidt, et al., 2008; Mozolic, Joyner, et al., 2008). Cross-modal deactivation therefore represents a likely mechanism by which selective attention interferes with multisensory integration and alters embodiment within the RHI as a function of attention instructions. Specifically, focusing on sight downplays somatosensations and prioritises information about the prosthesis, thereby yielding a stronger illusion. Conversely, attending to tactile sensations boosts the processing and the integration of somatosensory signals at the expense of the otherwise dominating visual sensory input (Sinnett et al., 2007). In sum, limited integration of visual information leads to a partial breakdown of the illusion—that is, visual information fails to override prior body signals and representations.

### The influence of cognitive load

Contrary to our original predictions, cognitive load hardly influenced the primary phenomenological components of interest. This outcome is consistent with a recent study that employed a similar approach to assess the role of working capacities in the context of the RHI (Fahey et al., 2018). While one could argue that our cognitive load manipulation insufficiently taxed cognitive resources or that this outcome stems from low statistical power given the modest effect size of cognitive load over multisensory integration (Buchan & Munhall, 2011a, 2012), evidence favoured the null hypothesis rather than indicating ambiguity, per Bayes factor analysis. Hence, working memory resources likely play a negligible role in the actual integration of visuotactile information in the context of the RHI (Fahey et al., 2018). Nevertheless, our cognitive load task was reliable in altering the affective component of the RHI, leading to lower ratings of pleasantness. This marginal effect may be due to the cognitive load interfering with the capacity to appraise the effect, thus leading participants to report lower pleasantness of the experience. An alternative, perhaps simpler explanation is that people usually prefer tasks that are easier, whereas the cognitive load component understandably makes the task more difficult and less pleasant.

Our results have theoretical and methodological implications. From a theoretical perspective, our present findings are consistent with, and extend, previous RHI studies (Fahey et al., 2018; Kilteni et al., 2015; Tsakiris, 2017). We also replicated previous research efforts concerned with the role of attentional processes in cross-modal sensory integration (Buchan & Munhall, 2011b; Déry et al., 2014; Fernández et al., 2015; Hartcher-O’Brien et al., 2017; Koelewijn et al., 2010; Lifshitz et al., 2013; Mozolic, Hugenschmidt, et al., 2008; Mozolic, Joyner, et al., 2008; Senkowski et al., 2005; Talsma et al., 2007, 2010; Talsma & Woldorff, 2005). Specifically, this study illustrates the importance for existing models of body ownership to accommodate and integrate attentional factors in developing a more comprehensive understanding of bodily self-consciousness. We also note that bodily self-consciousness differs from other forms of multisensory integration, as it relies, for example, on both somatosensory and external (visual) stimuli, rather than on purely exteroceptive stimuli (Blanke et al., 2015). From a methodological perspective, our results further support the influence of task instructions in the RHI. Subsequently, we encourage researchers to heed the importance of instructions and attention in this experimental approach.

### Limitations

Our study suffers from a few limitations. It is possible that our load manipulation insufficiently taxed cognitive resources, which would explain why this variable revealed no effect over the RHI, therefore rendering the interpretation of this experimental condition difficult. Moreover, the experimenter manually controlled exposure to experimental visual character strings, introducing variation in latency exposure across participants, which might have added an additional (though trivial) source of noise. Also, questions from the body ownership questionnaire followed a non-random order, which may have introduced an order effects bias. However, any such effects would likely affect all conditions in the same way, rendering them orthogonal to our experimental manipulations. Our factor structure was similar to previous work, which entails that order effects biases, if any, were negligible, and factors that included items presented later were not necessarily less reliable. Finally, recent work reveals that expectation significantly influence self-reports of body ownership distortions in the RHI, thereby indicating that demand characteristics are central to this experimental approach (Lush, 2020). While we cannot exclude this possibility here, demand characteristics were reported while contrasting synchronous and asynchronous stroking conditions. Due to our already packed two-by-two experimental design, we opted not to use asynchronous stroking as a baseline and instead focused our approach on relative differences between cognitive load and instructions manipulations. It therefore remains unclear how expectancy could have influenced the outcome of the present work, especially given that participants were blind to the purpose of our experimental manipulations.

Other limitations apply to our factor analysis procedures as well, as we drew our conclusions from a modest sample size and various explanatory components of a factor analysis approach, which obviously limits the generalisability of our findings (however, our average communalities and item:factor ratio were high). Pooling data from repeated measures and from the different conditions potentially limits the interpretability and generalisability of our conclusions by not directly accounting for between-group and within-subject variance (i.e., observations are not independent; Reise et al., 2005). Different experimental conditions could lead to slightly different phenomenological experiences, and though our collapsing of the data should average any such difference, this procedure contributes extra noise in the identification of factors (but also see the possibility of a Simpson’s paradox; Kievit et al., 2013). Factor scores distributed pseudo-normally and the determinant of the correlation matrix, the proportion of residuals greater than .05, and the root-mean-square residual fell outside recommended value ranges, which raises some concerns about the goodness-of-fit of the EFA. Moreover, a low determinant of the correlation matrix suggests multicollinearity, which can make it more difficult to determine the unique contribution of the correlated items to a given factor. However, this is less of a problem for factor analysis, unless it leads to Heywood cases. Overall, the implications of these values not meeting the highest diagnostic requirements are that it can limit the usefulness and interpretability of the model. Yet, models rarely meet these diagnostic requirements perfectly, thereby highlighting the benefits of model selection (Preacher et al., 2013). In addition, the impact of high multicollinearity in the data should prove to be minimal because the oblique rotation teases apart the different components and align those that correlate. Finally, our use of a different experimental design and additional items also limit strong comparisons to Longo et al. (2008). In this regard, our research effort primarily serves as a preliminary exploration.

## Conclusion

Unlike focusing on tactile sensations, attending to visual aspects of the rubber hand elicits a stronger illusion. Furthermore, increased cognitive load makes the RHI less enjoyable. Our study suggests that attention plays a central role in the RHI. Indeed, we found that task instructions regarding the attended modality influenced the strength of embodiment over the rubber hand. Specifically, emphasis on somatosensory sensations tends to weaken the overall experience compared to emphasis on visual sensations because instructions seem to decrease feelings of embodiment relative to the fake hand.

Our current findings have important implications for future research on multisensory integration and for studies employing RHI-like methodologies. First, these results contribute to our understanding of the role of attention in multisensory processes. Second, attention to particular features (e.g., visual vs. tactile aspects) may introduce considerable variation in body ownership. Thus, researchers should attempt to more fully account for attentional factors in existing models of body ownership. Third, and more specifically, participants may focus on one feature of the experience at the expense of another (e.g., proprioception or somatosensory sensations). Thus, it would behove researchers to provide explicit instructions emphasising visual representation, to maximise the illusory effect. In conclusion, our findings contribute to the debate over the role of top-down, higher-order cognitive factors in illusions of body ownership and multisensory integration.

## Supplemental Material

sj-pdf-1-qjp-10.1177_17470218221078858 – Supplemental material for The Rubber Hand Illusion: Top-down attention modulates embodiment Supplemental material, sj-pdf-1-qjp-10.1177_17470218221078858 for The Rubber Hand Illusion: Top-down attention modulates embodiment by Rémi Thériault, Mathieu Landry and Amir Raz in Quarterly Journal of Experimental Psychology

## Notes

1. Note, however, that a diminished illusion can still arise without synchronous stroking or tactile stimulation (e.g., Samad et al., 2015).

2. Note that there are known limitations to heartbeat detection tasks and mental tracking methods, such as low reliability with few samples or influence of extraneous factors (Brener & Ring, 2016; Kleckner et al., 2015).

3. While RHI experiments often include a control condition of asynchronous stroking as a baseline condition, we opted not to add this additional manipulation in our experiment. Given our two-by-two experimental design, additional baseline conditions would have doubled the number of conditions, to eight conditions in total, and made the analysis more difficult to interpret because it would have required to evaluate a three-way interaction. Furthermore, our experimental question concerned whether attentional factors can influence the illusion at its peak (i.e., during synchronous stroking), not in a weakened form (i.e., during asynchronous stroking), which would have therefore contributed information of limited utility.

4. The accuracy of character string reporting was not formally recorded. However, according to the experimenter, most if not all participants recalled the character string correctly, suggesting high accuracy and engagement with the task.

5. Here, we note that an oblique rotation should be used instead given the expected inter-factor correlations (Field et al., 2012).

6. We also refer the reader to discussions of demand characteristics, phenomenological control, and imaginative suggestion in the RHI (Dienes et al., 2020; Lush, 2020; Lush et al., 2020; Roseboom & Lush, 2020).
