# Supplementary material for: The Rubber Hand Illusion: Top-down attention modulates embodiment
Source: Q J Exp Psychol (Hove). 2022 Feb 24;75(11):2129–48. doi: 10.1177/17470218221078858 (PMC9516612; doi:10.1177/17470218221078858)
Supplement: sj-pdf-1-qjp-10.1177_17470218221078858 – Supplemental material for The Rubber Hand Illusion: Top-down attention modulates embodiment [file sj-pdf-1-qjp-10.1177_17470218221078858.pdf]

Supplementary Material for:

The Rubber Hand Illusion:

Top-Down Attention Modulates Embodiment

Rémi Thériault[a,b,c,*], Mathieu Landry[c,d], Amir Raz[a,c,e]

[a]Department of Psychiatry, McGill University

[b]Department of Psychology, Université du Québec à Montréal

[c]Institute for Interdisciplinary Brain and Behavioral Sciences, Chapman University

[d]Integrated Program in Neuroscience, Montreal Neurological Institute

[e]The Lady Davis Institute at the SMBD Jewish General Hospital

Author Note

*** Corresponding author:** Rémi Thériault, 100 rue Sherbrooke Ouest, Montréal, Québec, H2X 3P2, Canada. Email: remi.theriault@mail.mcgill.ca

Thériault, Landry, & Raz (2021)

Table S1

Factor Loadings/Pattern Matrix Comparison of Longo and Colleagues (2008) and Thériault, Landry and Raz (Current Paper) for Principal Component Analysis (PCA) with Orthogonal ("varimax") Rotation and Four Factors

**Synchronous condition (Longo and colleagues, 2008)**							**All 4 conditions (Thériault, Landry, & Raz)**						
**Item**	**During the block…**	**Embodiment of rubber hand**	**Loss of own hand**	**Movement**	**Affect**	**Communalities**	**Embodiment of rubber hand**	**Loss of own hand**	**Movement**	**Affect**	**Communalities**	**Item**	**During the block…**
1	…it seemed like I was looking directly at my own hand, rather than at a rubber hand.	0.817				0.698	0.899				0.872	2	I felt as if the hand I saw was my hand.
2	…it seemed like the rubber hand began to resemble my real hand.	0.747				0.675	0.536				0.548	6	It seemed like the hand I saw began to resemble my real hand.
3	…it seemed like the rubber hand belonged to me.	0.854				0.793	0.835				0.783	5	It seemed like the hand I saw belonged to me.
4	…it seemed like the rubber hand was my hand.	0.878				0.824	0.864				0.874	4	It seemed like the hand I saw was my hand.

Synchronous condition (Longo and colleagues, 2008)							All 4 conditions (Thériault, Landry, & Raz)						
**Item**	**During the block…**	**Embodiment of rubber hand**	**Loss of own hand**	**Movement**	**Affect**	**Communalities**	**Embodiment of rubber hand**	**Loss of own hand**	**Movement**	**Affect**	**Communalities**	**Item**	**During the block…**
5	…it seemed like the rubber hand was part of my body.	0.838				0.747	0.889				0.855	3	It seemed like the hand I saw was part of my body.
6	…it seemed like my hand was in the location where the rubber hand was.	0.733				0.655	0.833				0.797	16	It seemed like my hand was in the location where the hand I saw was.
7	…it seemed like the rubber hand was in the location where my hand was.	0.728				0.606	0.652				0.629	15	It seemed like the hand I saw was in the location where my hand was.
8	…it seemed like the touch I felt was caused by the paintbrush touching the rubber hand.	0.641				0.590	0.849				0.792	14	It seemed like the touch I felt was caused by the brush touching the hand I saw.

Synchronous condition (Longo and colleagues, 2008)							All 4 conditions (Thériault, Landry, & Raz)						
**Item**	**During the block…**	**Embodiment of rubber hand**	**Loss of own hand**	**Movement**	**Affect**	**Communalities**	**Embodiment of rubber hand**	**Loss of own hand**	**Movement**	**Affect**	**Communalities**	**Item**	**During the block…**
9	…it seemed like I could have moved the rubber hand if I had wanted.	0.651				0.542	0.875				0.796	7	It seemed like I could have moved the hand I saw if I had wanted.
10	…it seemed like I was in control of the rubber hand.	0.740				0.610	0.864				0.780	8	It seemed like I was in control of the hand I saw.
11	…it seemed like my own hand became rubbery.					0.457		0.564			0.522	32	It felt as if my (real) hand were turning 'rubbery'.
12	…it seemed like I was unable to move my hand.		0.700			0.628		0.657			0.647	28	It seemed like I was unable to move my hand.
13	…it seemed like I could have moved my hand if I had wanted.		-0.681			0.468		-0.698			0.545	29	It seemed like I could have moved my hand if I had wanted.

Synchronous condition (Longo and colleagues, 2008)							All 4 conditions (Thériault, Landry, & Raz)						
**Item**	**During the block…**	**Embodiment of rubber hand**	**Loss of own hand**	**Movement**	**Affect**	**Communalities**	**Embodiment of rubber hand**	**Loss of own hand**	**Movement**	**Affect**	**Communalities**	**Item**	**During the block…**
14	…it seemed like I couldn't really tell where my hand was.		0.575			0.487	0.580	0.552			0.656	18	It seemed like I couldn't really tell where my (real) hand was.
15	…it seemed like my hand had disappeared.		0.609			0.489	0.696	0.504			0.762	19	It seemed like my (real) hand had disappeared.
16	…it seemed like my hand was out of my control.		0.603			0.594		0.684			0.611	30	It seemed like my hand was out of my control.
17	…it seemed like my hand was moving towards the rubber hand.			0.747		0.617		0.511			0.482	17	I felt as if my (real) hand were drifting towards the left (towards the fake hand).

Synchronous condition (Longo and colleagues, 2008)							All 4 conditions (Thériault, Landry, & Raz)						
**Item**	**During the block…**	**Embodiment of rubber hand**	**Loss of own hand**	**Movement**	**Affect**	**Communalities**	**Embodiment of rubber hand**	**Loss of own hand**	**Movement**	**Affect**	**Communalities**	**Item**	**During the block…**
18	…it seemed like the rubber hand was moving towards my hand.			0.667		0.58					0.372	34	It appeared (visually) as if the fake hand was drifting to the right (towards my real hand).
19	…it seemed like I had three hands.			0.616		0.538			0.610		0.471	12	It felt as if I had two right hands.
20	I found that experience enjoyable.				0.840	0.724				0.734	0.756	20	I found the experience enjoyable.
21	I found that experience interesting.				0.618	0.427					0.355	21	I found the experience interesting.
22	…the touch of the paintbrush on my finger was pleasant.				0.755	0.643				0.866	0.830	22	The touch of the brush on my hand was pleasant.

Synchronous condition (Longo and colleagues, 2008)							All 4 conditions (Thériault, Landry, & Raz)						
**Item**	**During the block…**	**Embodiment of rubber hand**	**Loss of own hand**	**Movement**	**Affect**	**Communalities**	**Embodiment of rubber hand**	**Loss of own hand**	**Movement**	**Affect**	**Communalities**	**Item**	**During the block…**
23	…I had the sensation of pins and needles in my hand.					0.128		0.696			0.487	24	I had the sensation of pins and needles in my hand.
24	…I had the sensation that my hand was numb.					0.372		0.776			0.644	25	I had the sensation that my hand was numb.
25	…it seemed like the experience of my hands was less vivid than normal.					0.331		0.635			0.526	26	It seemed like the experience of my hands was less vivid than normal.
26	…I found myself liking the rubber hand.					0.524				0.646	0.687	23	I found myself liking the hand I saw.

Synchronous condition (Longo and colleagues, 2008)							All 4 conditions (Thériault, Landry, & Raz)						
**Item**	**During the block…**	**Embodiment of rubber hand**	**Loss of own hand**	**Movement**	**Affect**	**Communalities**	**Embodiment of rubber hand**	**Loss of own hand**	**Movement**	**Affect**	**Communalities**	**Item**	**During the block…**
27	…it seemed like I was feeling the touch of the paintbrush in the location where I saw the rubber hand being touched.					0.199	0.852				0.816	1	I felt the touch of the brush on the hand I saw.
								-0.554			0.602	10	I felt the touch of the brush on my (real) hand.
							0.561	0.567			0.642	11	It no longer felt like my (real) hand belonged to my body.
									0.721		0.664	13	I felt the touch of the brush on both hands at the same time.

**Synchronous condition (Longo and colleagues, 2008)**							**All 4 conditions (Thériault, Landry, & Raz)**						
**Item**	**During the block…**	**Embodiment of rubber hand**	**Loss of own hand**	**Movement**	**Affect**	**Communalities**	**Embodiment of rubber hand**	**Loss of own hand**	**Movement**	**Affect**	**Communalities**	**Item**	**During the block…**
									0.565		0.362	33	It seemed as if the touch I was feeling came from somewhere between my own hand and the fake hand.
											0.242	9	I felt like my right hand was colder than my left hand.
											0.268	27	The hand I saw was the hand of another person.
											0.300	31	I felt the touch of the brush on both hands, but never at the same time.

Synchronous condition (Longo and colleagues, 2008)							All 4 conditions (Thériault, Landry, & Raz)						
**Item**	**During the block…**	**Embodiment of rubber hand**	**Loss of own hand**	**Movement**	**Affect**	**Communalities**	**Embodiment of rubber hand**	**Loss of own hand**	**Movement**	**Affect**	**Communalities**	**Item**	**During the block…**
											0.178	35	The hand I saw began to resemble my own (real) hand, in terms of shape, skin tone, freckles or some other visual feature.
	Eigenvalues (SS loadings)	9.52	2.21	1.78	1.44		9.815	6.033	2.852	2.451			
	Percent variance explained (Proportion var)	26.3	12.2	9	7.8		28	17.2	8.1	7			

Note. Component loadings less than 0.5 are not displayed.

Grey = Question identical or equivalent to Longo.

Beige = Question related but not fully equivalent to Longo.

Dark Blue = Item loaded on a different dimension than Longo and colleagues’ (2008) original PCA.

Table S2

Factor Loadings/Pattern Matrix for Exploratory Factor Analysis (EFA) with Oblique Rotation and Four Factors (No Items Excluded)

Item	During the block…	Embodiment of rubber hand	Loss of own hand	Movement	Affect	Communalities
1	I felt the touch of the brush on the hand I saw.	0.863				0.806
2	I felt as if the hand I saw was my hand.	0.958				0.873
3	It seemed like the hand I saw was part of my body.	0.932				0.848
4	It seemed like the hand I saw was my hand.	0.882				0.876
5	It seemed like the hand I saw belonged to me.	0.839				0.768
6	It seemed like the hand I saw began to resemble my real hand.					0.523
7	It seemed like I could have moved the hand I saw if I had wanted.	0.92				0.775
8	It seemed like I was in control of the hand I saw.	0.902				0.759
9	I felt like my right hand was colder than my left hand.					0.164
10	I felt the touch of the brush on my (real) hand.			0.502		0.498
11	It no longer felt like my (real) hand belonged to my body.					0.614
12	It felt as if I had two right hands.					0.404
13	I felt the touch of the brush on both hands at the same time.			0.68		0.642
14	…it seemed like I couldn't really tell where my hand was.	0.863				0.778
15	It seemed like the hand I saw was in the location where my hand was.	0.599				0.583
16	It seemed like my hand was in the location where the hand I saw was.	0.835				0.783
17	I felt as if my (real) hand were drifting towards the left (towards the fake hand).		0.567			0.434
18	It seemed like I couldn't really tell where my (real) hand was.					0.638
19	It seemed like my (real) hand had disappeared.	0.664				0.761
20	I found the experience enjoyable.				0.691	0.688
21	I found the experience interesting.					0.296
22	The touch of the brush on my hand was pleasant.				0.946	0.872
23	I found myself liking the hand I saw.		0.514		0.557	0.601
24	I had the sensation of pins and needles in my hand.		0.641			0.412
25	I had the sensation that my hand was numb.		0.735			0.597
26	It seemed like the experience of my hands was less vivid than normal.		0.675			0.469
27	The hand I saw was the hand of another person.					0.135

28	It seemed like I was unable to move my hand.		0.754			0.617
29	It seemed like I could have moved my hand if I had wanted.		-0.675			0.489
30	It seemed like my hand was out of my control.		0.659			0.564
31	I felt the touch of the brush on both hands, but never at the same time.					0.174
32	It felt as if my (real) hand were turning ‘rubbery’.		0.506			0.482
33	It seemed as if the touch I was feeling came from somewhere between my own hand and the fake hand.					0.235
34	It appeared (visually) as if the fake hand was drifting to the right (towards my real hand).					0.283
35	The hand I saw began to resemble my own (real) hand, in terms of shape, skin tone, freckles or some other visual feature.					0.122
	Eigenvalues	9.892	5.650	1.885	2.135	
	Percent variance explained	28.3	16.1	5.4	6.1	

Note. Component loadings less than 0.5 are not displayed. Rows highlighted in red represent items that were excluded from the analysis reported in the manuscript.

Table S3

Correlation Matrix

	X1	X2	X3	X4	X5	X6	X7	X8	X9	X10	X11	X12	X13	X14	X15	X16	X17	X18	X19	X20	X21	X22	X23	X24	X25	X26	X27	X28	X29	X30	X31	X32	X33	X34	X35
X1	1.00	0.82	0.76	0.77	0.75	0.44	0.80	0.79	0.29	-0.30	0.51	0.37	0.48	0.82	0.61	0.72	0.31	0.56	0.62	0.50	0.48	0.23	0.40	0.04	0.07	0.15	-0.09	0.38	-0.25	0.47	0.28	0.37	0.17	0.18	0.16
X2	0.82	1.00	0.85	0.90	0.80	0.67	0.77	0.78	0.24	-0.35	0.60	0.32	0.35	0.82	0.61	0.84	0.36	0.65	0.71	0.32	0.40	0.01	0.43	0.21	0.30	0.23	-0.03	0.38	-0.26	0.45	0.16	0.47	0.15	0.27	0.28
X3	0.76	0.85	1.00	0.86	0.88	0.63	0.79	0.76	0.26	-0.34	0.63	0.28	0.36	0.76	0.62	0.81	0.38	0.63	0.70	0.35	0.49	0.04	0.38	0.19	0.27	0.24	-0.04	0.44	-0.29	0.46	0.19	0.49	0.09	0.26	0.21
X4	0.77	0.90	0.86	1.00	0.82	0.72	0.76	0.78	0.26	-0.45	0.64	0.33	0.29	0.79	0.62	0.81	0.45	0.68	0.75	0.31	0.40	0.03	0.49	0.23	0.34	0.32	-0.08	0.43	-0.30	0.46	0.23	0.53	0.15	0.37	0.31
X5	0.75	0.80	0.88	0.82	1.00	0.60	0.72	0.77	0.15	-0.34	0.56	0.28	0.33	0.76	0.59	0.77	0.38	0.58	0.64	0.39	0.50	0.14	0.46	0.15	0.26	0.23	-0.07	0.40	-0.29	0.39	0.24	0.46	0.17	0.28	0.24
X6	0.44	0.67	0.63	0.72	0.60	1.00	0.51	0.46	0.17	-0.37	0.50	0.21	0.18	0.49	0.49	0.59	0.43	0.47	0.53	0.21	0.28	-0.07	0.54	0.32	0.45	0.37	0.04	0.48	-0.37	0.44	0.09	0.48	0.12	0.42	0.53
X7	0.80	0.77	0.79	0.76	0.72	0.51	1.00	0.89	0.23	-0.28	0.47	0.25	0.42	0.73	0.59	0.74	0.28	0.53	0.59	0.44	0.46	0.18	0.35	0.06	0.14	0.23	-0.05	0.33	-0.12	0.39	0.17	0.37	0.03	0.19	0.23
X8	0.79	0.78	0.76	0.78	0.77	0.46	0.89	1.00	0.20	-0.25	0.50	0.28	0.38	0.77	0.59	0.73	0.30	0.52	0.61	0.40	0.41	0.16	0.39	0.13	0.22	0.22	-0.06	0.33	-0.13	0.40	0.21	0.39	0.01	0.21	0.22
X9	0.29	0.24	0.26	0.26	0.15	0.17	0.23	0.20	1.00	0.00	0.37	0.25	0.20	0.25	0.23	0.18	0.25	0.28	0.29	0.03	0.08	-0.12	0.11	0.13	0.22	0.22	-0.02	0.21	-0.20	0.20	0.08	0.39	0.11	0.26	0.03
X10	-0.30	-0.35	-0.34	-0.45	-0.34	-0.37	-0.28	-0.25	0.00	1.00	-0.47	0.01	0.13	-0.31	-0.04	-0.36	-0.20	-0.45	-0.49	0.05	-0.09	0.14	-0.28	-0.22	-0.35	-0.29	0.35	-0.24	0.43	-0.36	-0.35	-0.31	0.00	-0.03	-0.18
X11	0.51	0.60	0.63	0.64	0.56	0.50	0.47	0.50	0.37	-0.47	1.00	0.31	0.26	0.57	0.41	0.62	0.60	0.64	0.72	0.11	0.30	-0.13	0.37	0.38	0.47	0.36	-0.16	0.49	-0.42	0.50	0.29	0.51	0.13	0.30	0.12
X12	0.37	0.32	0.28	0.33	0.28	0.21	0.25	0.28	0.25	0.01	0.31	1.00	0.57	0.36	0.29	0.27	0.43	0.29	0.14	0.12	0.13	0.01	0.23	-0.01	0.11	0.47	0.02	0.36	-0.28	0.28	0.24	0.34	0.27	0.27	0.01
X13	0.48	0.35	0.36	0.29	0.33	0.18	0.42	0.38	0.20	0.13	0.26	0.57	1.00	0.45	0.49	0.35	0.31	0.26	0.11	0.35	0.29	0.14	0.26	0.01	0.01	0.30	0.19	0.36	-0.22	0.24	0.02	0.15	0.35	0.26	0.01
X14	0.82	0.82	0.76	0.79	0.76	0.49	0.73	0.77	0.25	-0.31	0.57	0.36	0.45	1.00	0.63	0.78	0.33	0.57	0.66	0.42	0.41	0.12	0.43	0.07	0.19	0.16	-0.02	0.40	-0.29	0.40	0.23	0.45	0.24	0.19	0.18
X15	0.61	0.61	0.62	0.62	0.59	0.49	0.59	0.59	0.23	-0.04	0.41	0.29	0.49	0.63	1.00	0.59	0.34	0.41	0.43	0.37	0.34	0.11	0.33	0.06	0.14	0.26	0.08	0.38	-0.24	0.29	0.02	0.37	0.29	0.43	0.25
X16	0.72	0.84	0.81	0.81	0.77	0.59	0.74	0.73	0.18	-0.36	0.62	0.27	0.35	0.78	0.59	1.00	0.45	0.70	0.77	0.33	0.41	0.09	0.40	0.25	0.32	0.30	-0.11	0.42	-0.30	0.48	0.18	0.47	0.20	0.21	0.23
X17	0.31	0.36	0.38	0.45	0.38	0.43	0.28	0.30	0.25	-0.20	0.60	0.43	0.31	0.33	0.34	0.45	1.00	0.51	0.47	0.16	0.16	0.03	0.40	0.28	0.39	0.39	-0.02	0.45	-0.33	0.40	0.15	0.41	0.29	0.46	0.05
X18	0.56	0.65	0.63	0.68	0.58	0.47	0.53	0.52	0.28	-0.45	0.64	0.29	0.26	0.57	0.41	0.70	0.51	1.00	0.81	0.21	0.30	0.04	0.44	0.41	0.45	0.49	-0.18	0.41	-0.33	0.59	0.32	0.42	0.18	0.23	0.17
X19	0.62	0.71	0.70	0.75	0.64	0.53	0.59	0.61	0.29	-0.49	0.72	0.14	0.11	0.66	0.43	0.77	0.47	0.81	1.00	0.22	0.35	0.02	0.41	0.36	0.46	0.33	-0.13	0.38	-0.33	0.57	0.31	0.48	0.09	0.19	0.19
X20	0.50	0.32	0.35	0.31	0.39	0.21	0.44	0.40	0.03	0.05	0.11	0.12	0.35	0.42	0.37	0.33	0.16	0.21	0.22	1.00	0.42	0.67	0.51	-0.11	-0.15	-0.11	0.04	0.20	0.09	0.13	0.13	0.04	0.17	0.10	0.03
X21	0.48	0.40	0.49	0.40	0.50	0.28	0.46	0.41	0.08	-0.09	0.30	0.13	0.29	0.41	0.34	0.41	0.16	0.30	0.35	0.42	1.00	0.28	0.32	0.13	0.07	0.09	0.09	0.22	-0.07	0.24	0.06	0.23	0.09	-0.02	0.22
X22	0.23	0.01	0.04	0.03	0.14	-0.07	0.18	0.16	-0.12	0.14	-0.13	0.01	0.14	0.12	0.11	0.09	0.03	0.04	0.02	0.67	0.28	1.00	0.41	-0.26	-0.32	-0.17	-0.12	0.01	0.32	-0.01	0.16	-0.12	-0.01	0.00	0.12
X23	0.40	0.43	0.38	0.49	0.46	0.54	0.35	0.39	0.11	-0.28	0.37	0.23	0.26	0.43	0.33	0.40	0.40	0.44	0.41	0.51	0.32	0.41	1.00	0.26	0.33	0.30	-0.01	0.42	-0.21	0.41	0.24	0.31	0.15	0.28	0.38
X24	0.04	0.21	0.19	0.23	0.15	0.32	0.06	0.13	0.13	-0.22	0.38	-0.01	0.01	0.07	0.06	0.25	0.28	0.41	0.36	-0.11	0.13	-0.26	0.26	1.00	0.68	0.36	0.14	0.42	-0.39	0.45	0.13	0.25	0.14	0.13	0.19
X25	0.07	0.30	0.27	0.34	0.26	0.45	0.14	0.22	0.22	-0.35	0.47	0.11	0.01	0.19	0.14	0.32	0.39	0.45	0.46	-0.15	0.07	-0.32	0.33	0.68	1.00	0.53	0.17	0.47	-0.44	0.41	0.17	0.45	0.16	0.31	0.18
X26	0.15	0.23	0.24	0.32	0.23	0.37	0.23	0.22	0.22	-0.29	0.36	0.47	0.30	0.16	0.26	0.30	0.39	0.49	0.33	-0.11	0.09	-0.17	0.30	0.36	0.53	1.00	0.05	0.48	-0.42	0.44	0.17	0.49	0.15	0.28	0.06
X27	-0.09	-0.03	-0.04	-0.08	-0.07	0.04	-0.05	-0.06	-0.02	0.35	-0.16	0.02	0.19	-0.02	0.08	-0.11	-0.02	-0.18	-0.13	0.04	0.09	-0.12	-0.01	0.14	0.17	0.05	1.00	0.12	0.11	-0.06	-0.16	-0.02	0.21	0.13	-0.05
X28	0.38	0.38	0.44	0.43	0.40	0.48	0.33	0.33	0.21	-0.24	0.49	0.36	0.36	0.40	0.38	0.42	0.45	0.41	0.38	0.20	0.22	0.01	0.42	0.42	0.47	0.48	0.12	1.00	-0.66	0.71	0.28	0.57	0.21	0.31	0.16
X29	-0.25	-0.26	-0.29	-0.30	-0.29	-0.37	-0.12	-0.13	-0.20	0.43	-0.42	-0.28	-0.22	-0.29	-0.24	-0.30	-0.33	-0.33	-0.33	0.09	-0.07	0.32	-0.21	-0.39	-0.44	-0.42	0.11	-0.66	1.00	-0.59	-0.23	-0.50	-0.30	-0.18	-0.10
X30	0.47	0.45	0.46	0.46	0.39	0.44	0.39	0.40	0.20	-0.36	0.50	0.28	0.24	0.40	0.29	0.48	0.40	0.59	0.57	0.13	0.24	-0.01	0.41	0.45	0.41	0.44	-0.06	0.71	-0.59	1.00	0.38	0.50	0.11	0.19	0.22
X31	0.28	0.16	0.19	0.23	0.24	0.09	0.17	0.21	0.08	-0.35	0.29	0.24	0.02	0.23	0.02	0.18	0.15	0.32	0.31	0.13	0.06	0.16	0.24	0.13	0.17	0.17	-0.16	0.28	-0.23	0.38	1.00	0.18	0.14	0.15	0.02
X32	0.37	0.47	0.49	0.53	0.46	0.48	0.37	0.39	0.39	-0.31	0.51	0.34	0.15	0.45	0.37	0.47	0.41	0.42	0.48	0.04	0.23	-0.12	0.31	0.25	0.45	0.49	-0.02	0.57	-0.50	0.50	0.18	1.00	0.13	0.38	0.20
X33	0.17	0.15	0.09	0.15	0.17	0.12	0.03	0.01	0.11	0.00	0.13	0.27	0.35	0.24	0.29	0.20	0.29	0.18	0.09	0.17	0.09	-0.01	0.15	0.14	0.16	0.15	0.21	0.21	-0.30	0.11	0.14	0.13	1.00	0.34	-0.02
X34	0.18	0.27	0.26	0.37	0.28	0.42	0.19	0.21	0.26	-0.03	0.30	0.27	0.26	0.19	0.43	0.21	0.46	0.23	0.19	0.10	-0.02	0.00	0.28	0.13	0.31	0.28	0.13	0.31	-0.18	0.19	0.15	0.38	0.34	1.00	0.17
X35	0.16	0.28	0.21	0.31	0.24	0.53	0.23	0.22	0.03	-0.18	0.12	0.01	0.01	0.18	0.25	0.23	0.05	0.17	0.19	0.03	0.22	0.12	0.38	0.19	0.18	0.06	-0.05	0.16	-0.10	0.22	0.02	0.20	-0.02	0.17	1.00

Table S4

Structure Matrix

	Embodiment of rubber hand	Loss of own hand	Two right hands	Affect
**X1**	0.8759542	0.26180714	0.34243708	0.4780727
**X2**	0.9221631	0.42232196	0.20099536	0.32201616
**X3**	0.9158357	0.42732969	0.20360879	0.34015382
**X5**	0.8722343	0.39968741	0.19911655	0.40411252
**X6**	0.6160115	0.59671949	0.11135561	0.26114571
**X7**	0.8639775	0.24714887	0.2551631	0.42213118
**X8**	0.8611762	0.28699242	0.24188196	0.39829003
**X10**	-0.3877578	-0.472248	0.24743828	0.08649364
**X11**	0.6615702	0.65586663	0.11676634	0.09439176
**X12**	0.3187751	0.30502186	0.69286516	0.10157817
**X13**	0.3985441	0.16344246	0.74168546	0.36545727
**X14**	0.875626	0.33014448	0.30350225	0.40103868
**X15**	0.656233	0.28718001	0.45185456	0.38839296
**X16**	0.8869607	0.47834684	0.16022954	0.30369133
**X17**	0.4169783	0.58481754	0.29925718	0.16222863
**X18**	0.6997568	0.63964987	0.06209941	0.169443
**X19**	0.7893734	0.61439241	-0.10657479	0.17414154
**X20**	0.4076565	-0.03766286	0.21775737	0.90059141
**X21**	0.4874807	0.16039025	0.11173001	0.42793002
**X23**	0.4548069	0.48970328	0.11786792	0.5578073
**X24**	0.1796839	0.6436696	-0.17773943	-0.05686079
**X25**	0.2744688	0.77144777	-0.10592061	-0.12357495
**X26**	0.2768932	0.65190132	0.30716384	-0.10354017
**X28**	0.4306056	0.72429015	0.33656337	0.19015851
**X29**	-0.2951049	-0.66203581	-0.17806059	0.10904764
**X30**	0.4978133	0.69990778	0.13742487	0.13291287
**X32**	0.498569	0.62721928	0.23176294	0.02169391
**X34**	0.2582153	0.37711906	0.33004634	0.1273316

Table S5

Hierarchical linear regression models for predicting the embodiment component as a function of load and instructions

Models	χ^2	*p*-value	BIC
Embodiment ~ β0 + S0 [Subject] + ε			331.05
Embodiment ~ β0 + S0[Subject] + *β1[Load]* + ε	.0008	*p* = .98	333.05
***Embodiment* ~ β0 + S0[Subject] + *β1[Load]* + *β2[Instructions]* + ε**	**21.39**	***p* < .001**	**313.66**
Embodiment ~ β0 + S0[Subject] + *β1[Load]* + *β2[Instructions]* + *β3[Load × Instructions]* + ε	2.23	*p* =.14	313.43

Note. Stepwise Chi-square goodness-of-fit values, corresponding *p*-values and Bayesian Criterion Information (BIC) of hierarchical linear regression models for predicting standardized embodiment scores across load (i.e., no load versus load), instructions (i.e., tactile versus visual), and their interactions as fixed factors, with participants as random factors. The best fitting model is in bold.

Table S6

Parameter estimates of best fitting hierarchical linear regression model for predicting the embodiment component as a function of load and instructions

Model: *Embodiment* ~ β0 + S0[Subject] + *β1[Load]* + *β2[Instructions]* + ε

Variables	Coefficient	Std. Error	95% C.I.
Intercept	-.25	.16	[-.57 .07]
Load	-.003	.1	[-.21 .2]
Instruction	.5	.1	[.29 .7]

Note. Parameter estimates, corresponding standard error, and 95% C.I. of best fitting hierarchical linear regression model for predicting standardized embodiment scores.

Table S7

Hierarchical linear regression models for predicting the feeling of having two right hands component as a function of load and instructions

Models	χ^2	*p*-value	BIC
***Two Right Hands* ~ β0 + S0 [Subject] + ε**			**356.4**
Two Right Hands ~ β0 + S0[Subject] + *β1[Load]* + ε	.3	*p* = .59	360.98
Two Right Hands ~ β0 + S0[Subject] + *β1[Load]* + *β2[Instructions]* + ε	.27	*p* = .6	365.59
Two Right Hands ~ β0 + S0[Subject] + *β1[Load]* + *β2[Instructions]* + *β3[Load × Instructions]* + ε	.63	*p* = .43	369.84

Note. Stepwise Chi-square goodness-of-fit values, corresponding *p*-values and Bayesian Criterion Information (BIC) of hierarchical linear regression models for predicting standardized feelings of two right hands scores across load (i.e., no load versus load), instructions (i.e., tactile versus visual), and their interactions as fixed factors, with participants as random factors. The best fitting model is in bold.

Table S8

Hierarchical linear regression models for predicting the loss of one's own hand component as a function of load and instructions

Models	χ^2	*p*-value	BIC
***Loss of Own Hand* ~ β0 + S0 [Subject] + ε**			**313.86**
Loss of Own Hand ~ β0 + S0[Subject] + *β1[Load]* + ε	.06	*p* = .8	318.33
Loss of Own Hand ~ β0 + S0[Subject] + *β1[Load]* + *β2[Instructions]* + ε	3.4	*p* = .07	319.82
Loss of Own Hand ~ β0 + S0[Subject] + *β1[Load]* + *β2[Instructions]* + *β3[Load × Instructions]* + ε	.23	*p* = .63	324.47

Note. Stepwise Chi-square goodness-of-fit values, corresponding *p*-values and Bayesian Criterion Information (BIC) of hierarchical linear regression models for predicting standardized feelings of loss own hand scores across load (i.e., no load versus load), instructions (i.e., tactile versus visual), and their interactions as fixed factors, with participants as random factors. The best fitting model is in bold.

Table S9

Hierarchical linear regression models for predicting the affect component as a function of load and instructions

Models	χ^2	*p*-value	BIC
Affect ~ β0 + S0 [Subject] + ε			298.86
***Affect* ~ β0 + S0[Subject] + *β1[Load]* + ε**	**4.27**	***p* < .05**	**299.12**
Affect ~ β0 + S0[Subject] + *β1[Load]* + *β2[Instructions]* + ε	1.56	*p* = .21	302.44
Affect ~ β0 + S0[Subject] + *β1[Load]* + *β2[Instructions]* + *β3[Load* × *Instructions]* + ε	1.1	*p* = .29	306.22

Note. Stepwise Chi-square goodness-of-fit values, corresponding *p*-values and Bayesian Criterion Information (BIC) of hierarchical linear regression models for predicting standardized feelings of loss own hand scores across load (i.e., no load versus load), instructions (i.e., tactile versus visual), and their interactions as fixed factors, with participants as random factors. The best fitting model is in bold.

Table S10

Parameter estimates of best fitting hierarchical linear regression model for predicting the affect component as a function of load

Model: *Affect* ~ β0 + S0[Subject] + *β1[Load]* + ε

Variables	Coefficient	Std. Error	95% C.I.
Intercept	.09	.17	[-.26 .44]
Load	-.17	.09	[-.35 -.009]

Note. Parameter estimates, corresponding standard error, and 95% C.I. of best fitting hierarchical linear regression model for predicting standardized embodiment scores.
